# Single-step Transepithelial photorefractive keratectomy in the treatment of mild, moderate, and high myopia: six month results

**DOI:** 10.1186/s12886-018-0888-x

**Published:** 2018-08-28

**Authors:** Lei Xi, Chen Zhang, Yanling He

**Affiliations:** 1 Department of Ophthalmology, Peking University International Hospital, Beijing, China; 2 Tianjin Medical University Eye hospital, Tianjin Medical University Eye Institute, School of Optometry and Ophthalmology, Tianjin, China; 3 Department of Ophthalmology, Peking University People’s Hospital, Xizhimen South Street 11, Xi Cheng District, Beijing, 100044 China

**Keywords:** Transepithelial photorefractive keratectomy, Myopia, TransPRK

## Abstract

**Background:**

To evaluate the safety, efficacy, and the refractive outcomes of single-step transepithelial photorefractive keratectomy (TransPRK) for the correction of mild, moderate, and high myopia.

**Methods:**

This study consecutively recruited 32 high myopic eyes, 32 mild myopic and 32 moderate myopic eyes. Eyes with myopia that had undergone TransPRK treatment. Pre- and post-operative visual and refractive data, corneal Higher Order Aberration (HOA) as well as safety and efficacy indices were analyzed at 6 months postoperatively.

**Results:**

Six months after TransPRK, the manifest refraction spherical equivalent (SE) was not significantly between high myopia group and moderate myopia group (*p* = 0.636). No eyes lost ≥2 lines of corrected distant visual acuity (CDVA) in high myopic eyes. The uncorrected distance visual acuity (UDVA) was significantly higher in low and moderate myopia groups than the high myopia group (*P* < 0.001; *P* = 0.002). The CDVA was not significantly different between moderate and high myopia groups (*P* = 0.057). There was no significant difference in mean safety index between high myopia group (1.01 ± 0.14) and mild myopia group (1.08 ± 0.15) (*P* > 0.05). The mean safety index was significantly higher in the moderate myopia group (1.16 ± 0.23) than in the high myopia group (1.01 ± 0.14) (*P* = 0.002). The efficacy index was significantly higher in the moderate myopia group (1.05 ± 0.20) than in the high myopia group (0.89 ± 0.17) (*P* = 0.02), and there was no significant difference between the high myopia group (0.89 ± 0.17) and the low myopia group (0.96 ± 0.16) (*P* = 0.14).

**Conclusions:**

The mean safety index was over 1.0 in the three groups. TransPRK showed acceptable safety and efficacy in the moderate myopic eyes, as well as mild and high myopic eyes. High myopic eyes got very similar refractive results with moderate myopic eyes six months postoperatively. The safety and efficacy indexes were not significantly different between the high myopia group and the low myopia group.

## Background

Transepithelial photorefractive keratectomy (TransPRK) is becoming increasingly popular in the treatment of myopia. TransPRK has a higher laser cutting frequency than traditional PRK. The unique feature of this technique is that it removes the corneal epithelium and stroma in a single step with one ablation profile. Its advantages include flap free, minimal trauma to the eye and without flap-related complications [1]. Moreover, the corneal biomechanics are less affected than other refractive procedures, including Small Incision Lenticule Extraction (SMILE) [2]. Also it allows reoperation. Previous studies have demonstrated that TransPRK is safe, predictable and effective in the correction of myopia and myopic astigmatism [3–6]. A study showed that TransPRK and femtosecond-assisted laser in situ keratomileusis (LASIK) share similar refractive outcomes in myopia correction [7]. Another study found that TransPRK using SmartPulse Technology (SPT) provides significant accelerated healing and visual rehabilitation than without SPT [8]. However, there is a lack of comparative data on the safety, efficacy and refractive outcomes between low to moderate myopic eyes and high myopic eyes after TransPRK surgery.

This prospective clinical study evaluated the early visual acuity, refractive error and efficacy outcomes of TransPRK in different ranges of myopic eyes with low (< 2D) astigmatism.

## Methods

### Patient population and study design

This study enrolled patients consecutively between October 2016 and March 2017 at the Department of Ophthalmology at Peking University. Patients were divided into three groups: low myopia (≤ − 3.00D), moderate myopia (− 3.00D to − 6.00D) and high myopia (≥ − 6.00 D) [9]. All the patients provided informed consent. The study followed the tenets of the Declaration of Helsinki and institutional review board.

### Patient enrolment criteria

Inclusion criteria were as follows: age over 18 years with stable refraction for at least 12 months, corrected distance visual acuity (CDVA) of at least 20/25, cylinder refraction lower than 2.0 diopter (D), discontinued contact lens use for at least 1 month, free of ocular disease and estimated postoperative corneal stromal bed thickness of more than 350 μm.

### Preoperative examination

Preoperative examination included slit-lamp examination, intraocular pressure measurement, corneal epithelium assessment by fluorescein staining, tear breakup time, Schirmer I test, UDVA and CDVA, corneal topography (Optikon SpA, Rome, ITALY), pentacam scheimpflug topography (Oculus, Wetzlar, Germany), manifest and cycloplegic refraction, ultrasound pachymetry and fundus examination.

### Surgical technique

All surgeries were performed by a single surgeon using the SCHWIND Amaris 500E excimer laser platform (SCHWIND eye-tech-solutions GmbH, Kleinostheim, Germany). The ablation algorithm was calculated using ORK-CAM software. For each treatment, the epithelium thickness profile that 55 μm centrally and 65 μm peripherally based on the population statistic. The target refraction was emmetropia in all eyes. After surgery, the cornea was irrigated with a cool balanced salt solution and a soft bandage contact lens was applied for three to four days. Patients were instructed to use 0.5% levofloxacin (Cravit; Santen, Inc.) four times a day for one week and 0.1% fluorometholone (Allergan, Inc.) eye drops four times a day, then tapered progressively over the following four months.

### Safety and efficacy

The safety index is defined as the ratio of postoperative CDVA/preoperative CDVA. The efficacy index is defined as the ratio of postoperative UDVA/preoperative CDVA.

### Corneal wavefront aberration measurement

Corneal wavefront aberration were measured by a rotating Scheimpflug Camera (Pentacam; Oculus). The examinations were made in a dark room in the morning. Higher order aberrations (HOAs) of the cornea with a 6.0-mm analysis diameter were calculated separately from the total cornea preoperatively and 6 months postoperatively.

## Statistical analysis

Data were analyzed using SPSS 20.0 (SPSS Inc., Chicago, IL, USA). The one-way analysis of variance (ANOVA) was used to compare the differences between the study groups. LSD was performed in the analysis. Differences with a *p* value of 0.05 or less were considered statistically significant. Pearson correlation test was used to analyze the correlation between the attempted SE refraction and the achieved SE refraction.

## Results

A total of 96 eyes were included in this study. Each group included 32 eyes. All eyes completed the six-month follow-up. The patients’ characteristics were shown in Table 1.

**Table 1:** Demographic characteristics of study patients

Group	Patients/eyes	Gender	Mean age	Age range
Low myopia	21/32	16 women, 5 men	30.76 ± 5.17	20–37
Moderate myopia	18/32	15 women, 3 men	29.11 ± 5.17	19–37
High myopia	21/32	11 women, 10 men	30.57 ± 4.43	23–38

Age is expressed as mean years±SD; Low myopia vs Moderate myopia *P* = 0.301; Low myopia vs High myopia *P* = 0.901; Moderate myopia vs High myopia *P* = 0.360

### Visual acuity

**Fig. 1:**
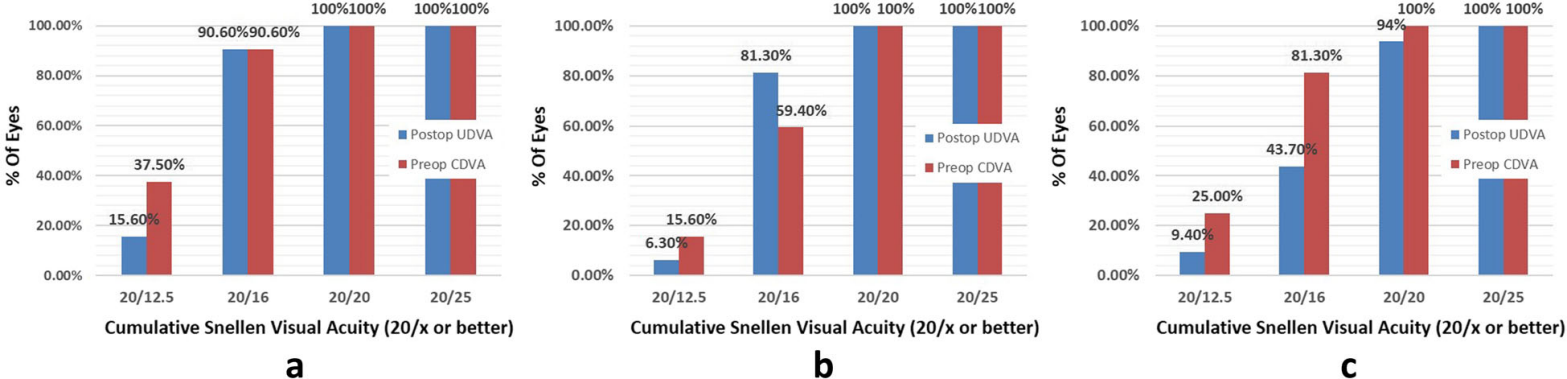
Cumulative percentage of eyes achieving uncorrected distance visual acuity (UDVA) 6 months postoperatively. (**a** mild; **b** moderate; **c** high)

**Fig. 2:**
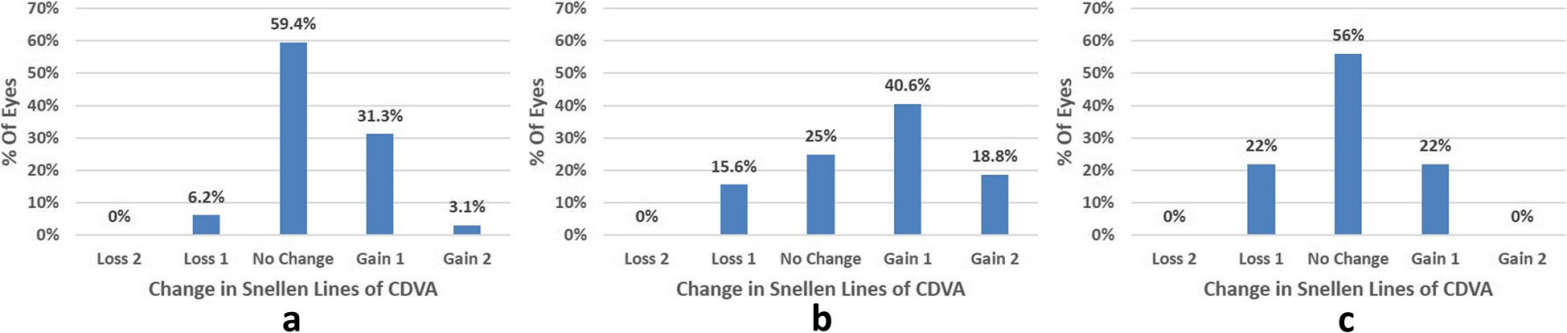
Changes in corrected distance visual acuity (CDVA) 6 months after TransPRK. (**a** mild; **b** moderate; **c** high)

Table 2 shows the preoperative variables of patients. The logMAR CDVA was not significantly different between the high myopia group and the low group (*P* = 0.198), and between the high group and the moderate group (*P* = 0.067). After six months, 100% of low myopia and moderate myopia had a UDVA of logMAR (20/20) or better, 94% of high myopia eyes had a UDVA of logMAR (20/20) or better (Fig. 1). There was no significant difference between the high myopia group and the moderate myopia group in the CDVA (*P* = 0.057) (Table 3). The best corrected visual acuity of patients with low myopia, moderate myopia and high myopia is greater than logMAR (20/20). Figure 2 shows the change of Snellen lines of logMAR CDVA. No eye lost 2 or more lines of CDVA.

**Table 2:** Preoperative Variables of Patients

	Low myopia	Moderate myopia	High myopia		
	(Mean ± SD)	(Mean ± SD)	(Mean ± SD)	*F (P)*	*P*
					*<0.001
Sphere (D)	−1.25 to −3.00	−3.25 to −5.50	−6.00 to −7.50	349.29 (<0.001)	^†^<0.001
	−2.43 ± 0.57	−4.16 ± 0.72	−6.39 ± 0.50		^‡^<0.001
					*0.296
Cylinder (D)	0.00 to −1.75	0.00 to − 1.75	0.00 to − 1.75	2.332 (0.103)	^†^0.27
	−0.70 ± 0.46	−0.56 ± 0.54	− 0.88 ± 0.58		^‡^0.033
					*0.02
SE refraction (D)	−1.25 to −3.625	−3.25 to −6.25	−6.00 to −8.00	262.51 (<0.001)	^†^<0.001
	−2.78 ± 0.65	−4.43 ± 0.85	−6.85 ± 0.61		^‡^<0.001
					*<0.001
UDVA (logMAR)	0.30 to 1.30	0.70 to 1.50	0.80 to 1.50	32.22 (<0.001)	^†^<0.001
	0.77 ± 0.21	0.97 ± 0.19	1.18 ± 0.20		^‡^<0.001
					*0.002
CDVA (logMAR)	0.00 to −0.20	0.00 to −0.20	0.00 to −0.20	5.01 (<0.001)	^†^0.198
	−0.128 ± 0.063	−0.075 ± 0.072	− 0.103 ± 0.07		^‡^0.067

SE = spherical equivalent refraction, UCVA = uncorrected visual acuity, CDVA = corrected distance visual acuity

Data are expressed as means±SD. *Low myopia vs Moderate myopia. ^†^Low myopia vs High myopia. ^‡^Moderate myopia vs High myopia

**Table 3:** Postoperative Variables of Patients

	Low myopia	Moderate myopia	High myopia		
	(Mean ± SD)	(Mean ± SD)	(Mean ± SD)	*F (P)*	*P*
					*<0.001
Sphere (D)	0.00 to − 0.75	−0.75 to 1.25	− 0.50 to 1.00	12.10 (<0.001)	^†^<0.001
	−0.32 ± 0.23	0.19 ± 0.54	0.28 ± 0.51		^‡^0.406
					*0.705
Cylinder (D)	0.00 to −0.75	0.00 to −1.00	0.00 to −1.00	0.98 (0.378)	^†^0.079
	−0.32 ± 0.23	−0.34 ± 0.23	− 0.43 ± 0.27		^‡^0.196
					*0.015
SE refraction (D)	−0.25 to 1.125	−0.875 to 1.00	−1.00 to 0.875	3.45 (0.036)	^†^0.048
	0.30 ± 0.33	0.02 ± 0.51	0.07 ± 0.52		^‡^0.636
					*0.408
UDVA (logMAR)	−0.20 to 0.00	−0.20 to 0.00	−0.20 to 0.10	8.65 (<0.001)	^†^<0.001
	−0.106 ± 0.05	−0.09 ± 0.05	− 0.047 ± 0.076		^‡^0.002
					*0.18
CDVA (logMAR)	−0.20 to 0.00	−0.20 to 0.00	− 0.20 to 0.00	5.43 (0.006)	^†^0.001
	−0.159 ± 0.056	−0.138 ± 0.066	− 0.106 ± 0.072		^‡^0.057

SE = spherical equivalent refraction, UCVA = uncorrected visual acuity, CDVA = corrected distance visual acuity.Data are expressed as Means±SD. *Low myopia vs Moderate myopia. ^†^Low myopia vs High myopia. ^‡^Moderate myopia vs High myopia

### Refractive results and accuracy

**Fig. 3:**
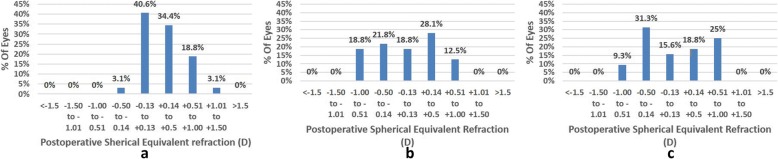
Percentage of eyes achieving various ranges of SE 6 months after TransPRK. (**a** mild; **b** moderate; **c** high)

**Fig. 4:**
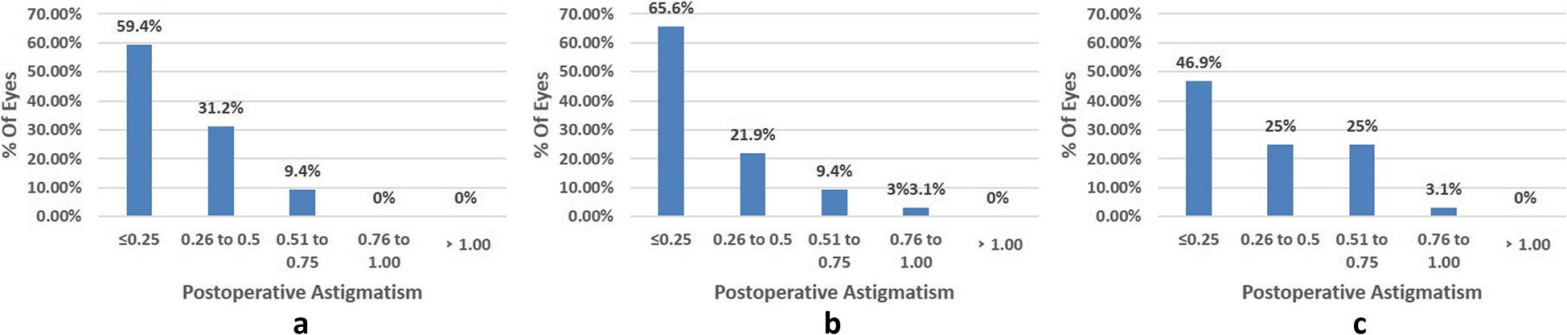
Percentage of eyes achieving various ranges of astigmatism 6 months after TransPRK. (**a** mild; **b** moderate; **c** high)

Table 3 shows the postoperative refraction. The spherical equivalent refraction (SE) was not significantly different between the high myopia group and the moderate myopia group (*P* = 0.636). The postoperative UDVA was lower in the high myopia group than in low to moderate myopia groups (*P* < 0.001; *P* = 0.002). The postoperative SE was shown in Fig. 3. 65.7% of eyes had SE within ±0.50D in the high myopia group, 78.1% and 87.5% in the low and moderate myopia groups. After 6 months, 90.6% of eyes had between 0.00 and 0.50D of astigmatism in the low myopia group, as compared with 87.5% in the moderate group and 71.9% in the high myopia group (Fig. 4).

**Fig. 5:**
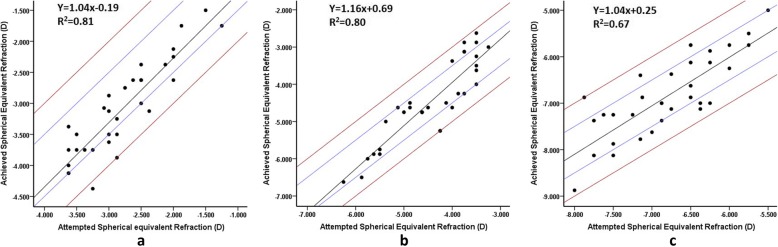
Achieved versus attempted spherical equivalent corrections 6 months postoperatively (**a** mild; **b** moderate; **c** high)The black line indicates the outcome of linear regression analysis, the area between two blue lines mean within ±0.50D

The correlation between attempted and achieved SE datas were shown in Fig. 5 (R^2^ = 0.81 for low myopia, R^2^ = 0.80 for moderate myopia and R^2^ = 0.67 for high myopia). 4% of the eyes were overcorrected in the low myopia and the high myopia group. 16% of the eyes were overcorrected in the moderate myopia group.

### Safety and efficacy

**Fig. 6:**
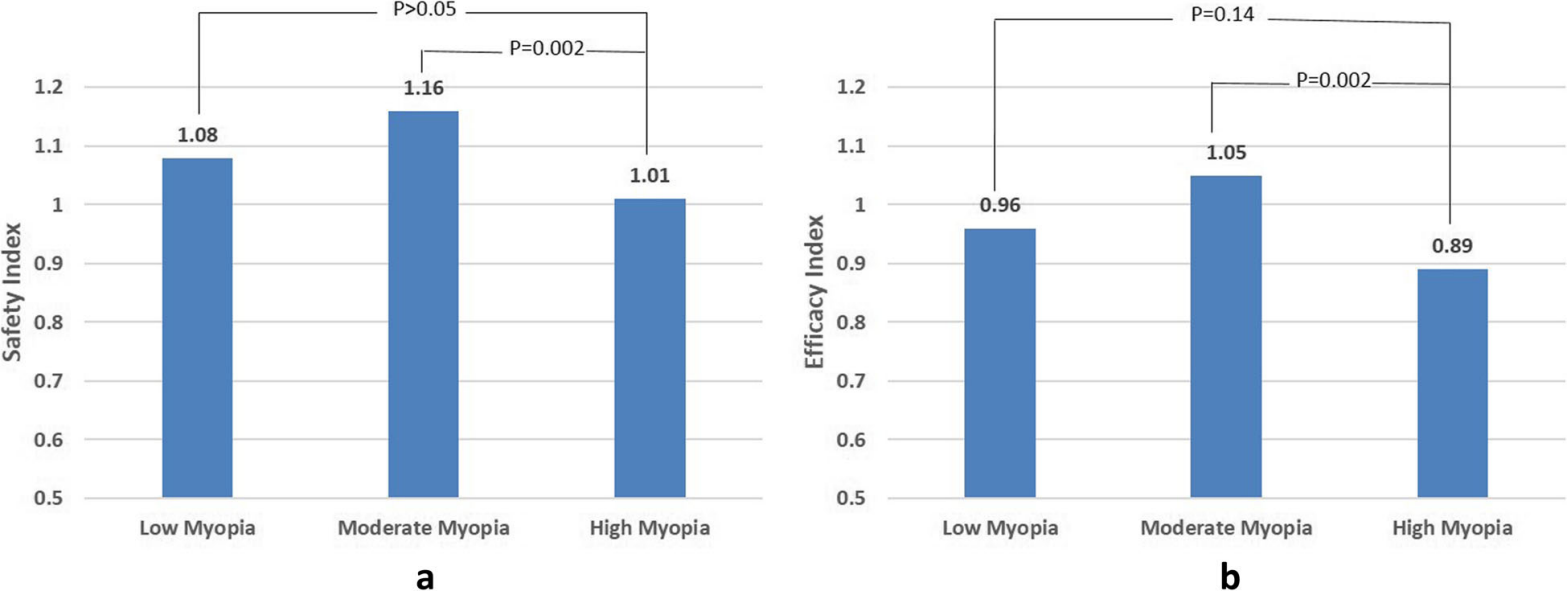
**a** Safety index (ratio of postoperative CDVA/preoperative CDVA). **b** Efficacy index (ratio of postoperative UDVA/preoperative CDVA)

The mean safety index was over 1.0 in the three groups (Fig. 6). The safety index was not significantly different between the high myopia group (1.01 ± 0.14) and the low myopia group (1.08 ± 0.15) (*P* > 0.05). The moderate myopia group (1.16 ± 0.23) was significantly higher than the high myopia group (*P* = 0.002).

The efficacy index was 0.96 ± 0.16 in the low myopia group, 1.05 ± 0.20 in the moderate myopia group, and 0.89 ± 0.17 in the high myopia group. The differences in efficacy index between the high myopia group and the low myopia were not statistically significant (*P* = 0.14). However, the moderate myopia group was significantly higher than high myopia group (*P* = 0.002).

### Corneal HOAs

The preoperative Corneal HOAs were not significantly different between the three groups. After six months, Table 4 showed that the high myopic corneal HOAs (1.07 ± 0.26) were significantly higher than low myopic corneal HOAs (0.64 ± 0.20) (*P*<0.001) and moderate myopic corneal HOAs (0.75 ± 0.20) (*P* < 0.001).

**Table 4:** Summary of corneal HOAs preoperatively and six months postoperatively

	Preoperation	*F (P)*	*P*	Postoperation	*F (P)*	*P*
Low myopia	0.39 ± 0.11	2.18 (0.119)	*0.808	0.64 ± 0.20	33.21 (<0.001)	*0.058
Moderate myopia	0.40 ± 0.12		^†^0.058	0.75 ± 0.20		^†^<0.001
High myopia	0.45 ± 0.14		^‡^0.098	1.07 ± 0.26		^‡^<0.001

Data are expressed as Means±SD. *Low myopia vs Moderate myopia. ^†^Low myopia vs High myopia. ^‡^Moderate myopia vs High myopia

## Discussion

This study demonstrated that one-step TransPRK could correct low to high myopia effectively. Six months after surgery, there was a significant improvement in UDVA, SE and astigmatism in the low, moderate and high myopia groups. More than 95% of the treated eyes were within ±1.00D of the intended target refraction. No eye lost two or more lines of CDVA.

Nearly 80% of the eyes in the low and moderate myopia groups and 65% of eyes in the high myopia group reached within ±0.50D of SE by six months after the operation. Previous clinical studies [3, 4, 10–13] have reported acceptable visual and refractive outcomes after TransPRK. However, most of these studies concentrated on low and moderate myopia or high myopia only. In this study, we analyzed our results in different groups of myopia.

In our study, 100% of low and moderate myopia eyes achieved a UDVA of 20/20 or better six months after the operation, while 94% of the high myopia eyes achieved a UDVA of 20/20 or better. Our results are comparable to the previous studies of TransPRK [3, 5, 7] and small-incision lenticule extraction. [14, 15] We found a statistically significant difference in postoperative UDVA between the low and moderate myopia groups versus the high myopia group. The reason may be the increased HOAs of the cornea postoperatively or the changes of high myopia fundus preoperatively. However, there was no significant difference in the CDVA between the moderate myopia group and the high myopia group. This indicated that TransPRK for high myopia was safe.

In our high myopia group, 65.7% of eyes were within ±0.50D and 100% of eyes were within ±1.0D of the intended SE refraction six months postoperatively. Our results agreed to some extent with other studies. Antonios et al. [3] found that 81.3% and 96.6% were within ±0.50D and ± 1.0D in high myopia patients 12 months postoperatively. Aslanides et al. [13] reported 91.4% and 97.1% were within ±0.50D and ± 1.0D by using Mitomycin C (MMC) therapy for the prevention of haze. They got accurate results than us within ±0.50D. There were no significant differences in the SE within ±1.0D. We found a difference between the attempted and the achieved SE correction in the three groups, with a tendency of overcorrection. The overcorrection may be related to corneal dehydration during surgery. The longer time possibly increases dehydration of the corneal stroma [1]. We suppose that the ablation of TransPRK should be modified in our future work.

In terms of safety, the mean safety index was greater than 1.0 in the three groups. The highest safety index was seen in the moderate myopia group in our study. In the low myopia group, 93.8% of eyes had no change or better CDVA postoperatively. In the moderate myopia group, 15.6% of eyes lost one line of CDVA and more than a half of eyes gained one or two lines of CDVA postoperatively. While in high myopia group, 22% of eyes lost one line CDVA. However, no statistically significant difference was found in the postoperative CDVA between the moderate and high myopia groups. The loss of the BCVA may be caused by the increase of the HOAs on the cornea postoperatively. Our results are more or less similar to other studies of refractive surgeries. Antonios R et al. [3] reported that 81.3% of high myopia eyes were between±0.50D after the treatment of TransPRK. Serrao S et al. [16] reported the safety index of the high myopia eyes treated by PRK was 0.81 one year postoperatively. Ikeda T et al. [17] found 77% of high myopic eyes showed no change or gain in CDVA one year after LASIK. Torky MA et al. [14] found that 88.2% of high myopic eyes got the SE within±0.50D by SMILE surgery six months postoperatively. Similarly, Jin HY et al. [18] found that 87% of high myopic eyes got the SE within±0.50D by SMILE surgery .

Moreover, the efficacy and UDVA were improved significantly in each group. The highest efficacy index was seen in the moderate myopia group. No differences in efficacy were found between the high myopia group and the low myopia group. The study indicates that TransPRK is effective for moderate myopia, as well as mild and high myopia. The single-step ablation profile targets 55 μm centrally and 65 μm peripherally, using theoretical simulations for the scope of ablation optical zone (OZ). Different patients showed different corneal epithelial thicknesses. Mild myopia patients may be more influenced by the difference between the surgical setting of corneal thickness and actual corneal thickness.

Corneal HOA changes were evaluated in this study. We found a significant increase in total corneal HOAs after surgery. Previous studies had reported that HOAs were related to the shadows, halos and night vision glare [19, 20]. The high myopia group showed significantly higher corneal HOAs than the low and moderate myopia groups. One study found that an RMS value of HOAs less than 1.0 had no noticeable effect on the clarity of retinal image, while blur could be seen with 1.0 to 1.5 μm of wavefront aberrations [21]. This may cause the decreased CDVA and UDVA in high myopia group postoperatively.

In conclusion, our data shows that TransPRK is a safe and effective surgical option in mild to high myopia. A large sample size and long-term results are needed in furture studies.

## Conclusions

TransPRK is a safe and effective surgical option in the treatment of mild and moderate myopia, and showed acceptable safety and efficacy in high myopia.

## Abbreviations

CDVA: corrected distance visual acuity
HOAs: higher order wavefront aberrations
LASIK: laser in situ keratomileusis
MMC: Mitomycin C
OZ: optical zone
SE: spherical equivalent
SPT: SmartPulse Technology
TransPRK: Transepithelial photorefractive keratectomy
UDVA: uncorrected distance visual acuity
